# A Cascade of Wnt, Eda, and Shh Signaling Is Essential for Touch Dome Merkel Cell Development

**DOI:** 10.1371/journal.pgen.1006150

**Published:** 2016-07-14

**Authors:** Ying Xiao, Daniel T. Thoresen, Lingling Miao, Jonathan S. Williams, Chaochen Wang, Radhika P. Atit, Sunny Y. Wong, Isaac Brownell

**Affiliations:** 1 Dermatology Branch, Center of Cancer Research, National Cancer Institute, National Institutes of Health, Bethesda, Maryland, United States of America; 2 Laboratory of Genetics and Physiology, National Institute of Diabetes and Digestive and Kidney Diseases, National Institutes of Health, Bethesda, Maryland, United States of America; 3 Department of Biology, Case Western Reserve University, Cleveland, Ohio, United States of America; 4 Departments of Dermatology and Cell and Developmental Biology, University of Michigan, Ann Arbor, Michigan, United States of America; University of Bradford, UNITED KINGDOM

## Abstract

The Sonic hedgehog (Shh) signaling pathway regulates developmental, homeostatic, and repair processes throughout the body. In the skin, touch domes develop in tandem with primary hair follicles and contain sensory Merkel cells. The developmental signaling requirements for touch dome specification are largely unknown. We found dermal Wnt signaling and subsequent epidermal Eda/Edar signaling promoted Merkel cell morphogenesis by inducing Shh expression in early follicles. Lineage-specific gene deletions revealed intraepithelial Shh signaling was necessary for Merkel cell specification. Additionally, a Shh signaling agonist was sufficient to rescue Merkel cell differentiation in Edar-deficient skin. Moreover, Merkel cells formed in Fgf20 mutant skin where primary hair formation was defective but Shh production was preserved. Although developmentally associated with hair follicles, fate mapping demonstrated Merkel cells primarily originated outside the hair follicle lineage. These findings suggest that touch dome development requires Wnt-dependent mesenchymal signals to establish reciprocal signaling within the developing ectoderm, including Eda signaling to primary hair placodes and ultimately Shh signaling from primary follicles to extrafollicular Merkel cell progenitors. Shh signaling often demonstrates pleiotropic effects within a structure over time. In postnatal skin, Shh is known to regulate the self-renewal, but not the differentiation, of touch dome stem cells. Our findings relate the varied effects of Shh in the touch dome to the ligand source, with locally produced Shh acting as a morphogen essential for lineage specification during development and neural Shh regulating postnatal touch dome stem cell maintenance.

## Author Summary

Sonic hedgehog (Shh) is one of a limited set of signaling molecules that cells use to drive organ formation during development and tissue regeneration after birth. How Shh signaling achieves different biological effects in the same tissue is incompletely understood. Touch domes are unique sensory structures in the skin that contain innervated Merkel cells. Using mouse genetics, we show that touch domes develop in tandem with, but distinct from, primary hair follicles. Moreover, touch dome specification requires a cascade of cell-cell signaling that ends with Shh signaling from an adjacent primary hair follicle. It was previously shown that Shh signaling from sensory nerves regulates the maintenance of touch dome stem cells after birth. Thus, the critical role for Shh signaling in embryonic touch dome specification is dependent on locally produced Shh, whereas the renewal of touch dome stem cells requires Shh transported to the skin by sensory neurons. These observations suggest that the distinct functions of Shh in touch dome development and maintenance correspond to changes in the source of the Shh signal required for the varied effects.

## Introduction

The Hedgehog (Hh) pathway is conserved across the Metazoa subkingdom, and is one of a small number of intercellular signaling pathways that regulate the differentiation and pattering of morphologically diverse structures during development [1,2]. Postnatally, Hh ligands regulate tissue specific stem cell, homeostasis, and wound healing [3]. The basic molecular mechanisms of Hh signaling are still being investigated, and even less is known about how activation the pathway can result in such pleiotropic functions. Timing and distribution of Hh ligand delivery, ligand concentration, and duration of exposure can all influence signaling outcomes [4]. Multiple additional mechanisms have been proposed to alter Hh signaling, including ligand modification and sequestration, regulation of the primary cilium, modulation of Smo function by kinases, redundancy and cross regulation of Gli transcription factors, altering target gene expression by transcriptional co-regulators, and cross regulation by other signaling pathways [1–5]. In the present study, we discovered that Shh is the final critical element in a signaling cascade that specifies the touch dome lineage in developing mouse skin. Contrasting these findings with the role of Shh in regulating postnatal touch dome stem cells [6], we found the changing function of Shh was accompanied by a change in the source of the ligand, suggesting an additional contextual mechanism that influences the results of Shh signaling.

The functional diversity of vertebrate skin depends greatly on the variety of ectodermal appendages it produces. The development of ectodermal appendages including hair follicles, teeth, sweat glands, and mammary glands is precisely regulated by networks of signaling pathways including Wnt/β-catenin, Eda/Edar, Shh, and BMP [7]. Hair follicle development is particularly well studied as a model of ectodermal appendage development [8]. Identifying and comparing the developmental networks that control the specification and differentiation of ectodermal lineages can provide insights into developmental disorders and genetic diseases.

The touch dome (TD) is a specialized epidermal sensory structure composed of K8+ Merkel cells (MCs) arrayed among columnar basal keratinocytes that express the hair follicle keratin K17. Cells of the TD are morphologically and molecularly distinct from the adjacent interfollicular epidermis. The TD arises from the K14+ ectoderm [9,10] and is maintained as a distinct epidermal lineage by resident stem cells [6,11–13]. In postnatal skin, self-renewal of TD stem cells is regulated by Shh from sensory neurons that innervate the MCs [6]. MC development requires the transcription factor Atoh1 [14] and is regulated by levels of Sox2 expression [15,16]. TD MCs can be identified in the epidermis based on their expression of Atoh1, Sox2, or K8 [17].

**Fig 1:**
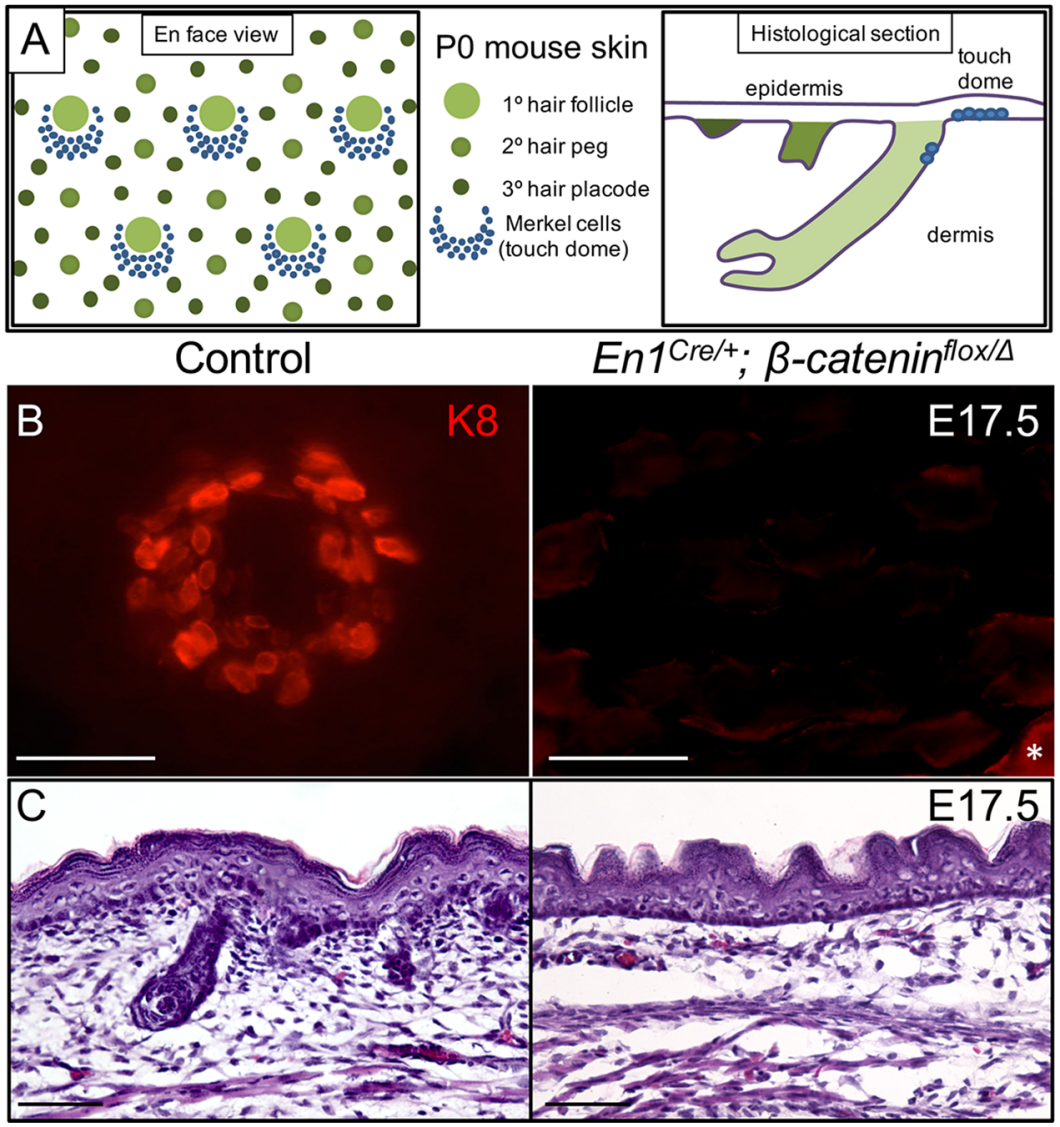
Dermal β-catenin is required for TD MC formation. (A) Schematic diagram of P0 mouse skin illustrating the relationship of TD MCs to primary hair follicles viewed en face from above the skin surface and on parasagittal section of dorsal trunk skin. (B) En face image of K8 whole mount staining in control (*En1*^*Cre/+*^*; β-catenin*^*flox/+*^) and *En1*^*Cre/+*^*; β-catenin*^*flox/Δ*^ dorsal trunk skin at E17.5. *, staining in the periderm. (C) H&E staining in control and *En1*^*Cre/+*^*; β-catenin*^*flox/Δ*^ mouse dorsal trunk skin at E17.5. Scale bars, 50 μm.

TD MC development in the embryonic ectoderm is spatially and temporally associated with that of primary hair follicles. In embryonic day 14 (E14) mice, a primary wave of hair follicle placode induction takes place. These primary follicles produce guard hairs that ultimately comprise ~2% of the adult mouse coat. Secondary (E16) and tertiary (E18) waves of hair follicle induction are responsible for forming the three remaining types of hair follicles [18]. Developing MCs are first detected at ~E15 in association with nascent primary hair germs. Just before birth, TD MCs surround the infundibulum at the top of guard hair follicles. Shortly after birth, the touch dome has migrated into a crescent-shaped domain just caudal to the guard hair follicle (Fig 1A). Planar cell polarity signals regulate the reorganization of the forming TD [19]. Loss of Eda function results in abnormal guard hair formation and an absence of TD MCs, however the mechanism by which Eda signaling impacts MC specification is unclear [20]. Other signals involved in TD MC development are largely unknown.

To elucidate the developmental requirements for TD MC formation, we used genetically modified mice to disrupt signaling pathways known to be important in the development of other ectodermal appendages. We found that embryonic deletion of dermal β-catenin prevented TD MC formation. Similarly, Edar mutant skin failed to generate TD MCs. The mechanism of MC loss in these mice was due to failure of Shh expression by primary hair follicles, as evidenced by an absence of TD MC formation in Shh-null skin. Moreover, independent deletion of *Shh* or *Smo* in the embryonic epidermis reveled that intraepithelial Shh signaling from primary hair germs was necessary for TD MC specification. Notably, the loss of MC specification in Shh-deficient skin was observed before any disruption in hair follicle development was apparent. The importance of Shh signaling in TD MC formation was further demonstrated by using a Smo agonist to rescue MC specification in *ex vivo*-cultured Edar mutant skin. In contrast, Fgf20 was dispensable in TD MC development, demonstrating that the signaling cascades required for guard hair follicle development and TD formation diverge at the level of Fgf20 signaling. Distinction from the forming guard hair follicle was further demonstrated when fate mapping of the follicle lineage showed that TD MC progenitors predominantly arise in the epidermis outside the hair placode. Thus, like other ectodermal appendages, the touch dome is a distinct epidermal lineage whose specification and development requires Wnt-dependent mesenchymal-epithelial interactions and reciprocal signaling within the developing ectoderm, including Eda signaling to primary hair placodes and subsequent Shh production by primary hair germs. The critical role for Shh signaling in embryonic TD specification is dependent on locally produced ligand, whereas the regulation of postnatal TD stem cells requires Shh transported to the skin by sensory neurons. These observations suggest that ligand source influences the differential effects of Shh signaling in the TD.

## Results

### Dermal β-Catenin Is Required for Touch Dome Morphogenesis

Mesenchymal-epithelial interaction is critical in hair follicle morphogenesis [21]. In mice with dermal β-catenin conditional knockout, hair follicle development is arrested at a very early stage [22]. We investigated the effect of dermal β-catenin deletion on TD MC development using *En1*^*Cre/+*^*; β-catenin*^*flox/Δ*^ mouse embryos where Cre recombination occurs in the dorsal trunk mesenchyme that forms the dermis [22]. As expected, dorsal trunk skin of E17.5 affected mice had no evidence of developing hair follicles on histological sections (Fig 1C). Using whole mount K8 immunostaining to detect MCs, we found K8+ TD MC adjacent to developing guard hair follicles in dorsal trunk skin of control (*En1*^*Cre/+*^*; β-catenin*^*flox/+*^, n = 5) E17.5 embryos (Fig 1B). In contrast, we found no K8 staining in dorsal trunk epidermis of E17.5 *En1*^*Cre/+*^*; β-catenin*^*flox/Δ*^ embryos (n = 3, Fig 1B), indicating that β-catenin function in the developing dermis is necessary for the formation of TD MCs. *En1*^*Cre*^ is not expressed in proximal limb mesenchyme [23]. As an internal control, we examined limb skin of E17.5 *En1*^*Cre/+*^*; β-catenin*^*flox/Δ*^ embryos and observed normal collections of K8+ MCs in TDs adjacent to hair follicles, further implicating dermal Wnt/β-catenin signaling in TD MC development.

### Epidermal Edar Function Is Necessary for Touch Dome Merkel Cell Formation

**Fig 2:**
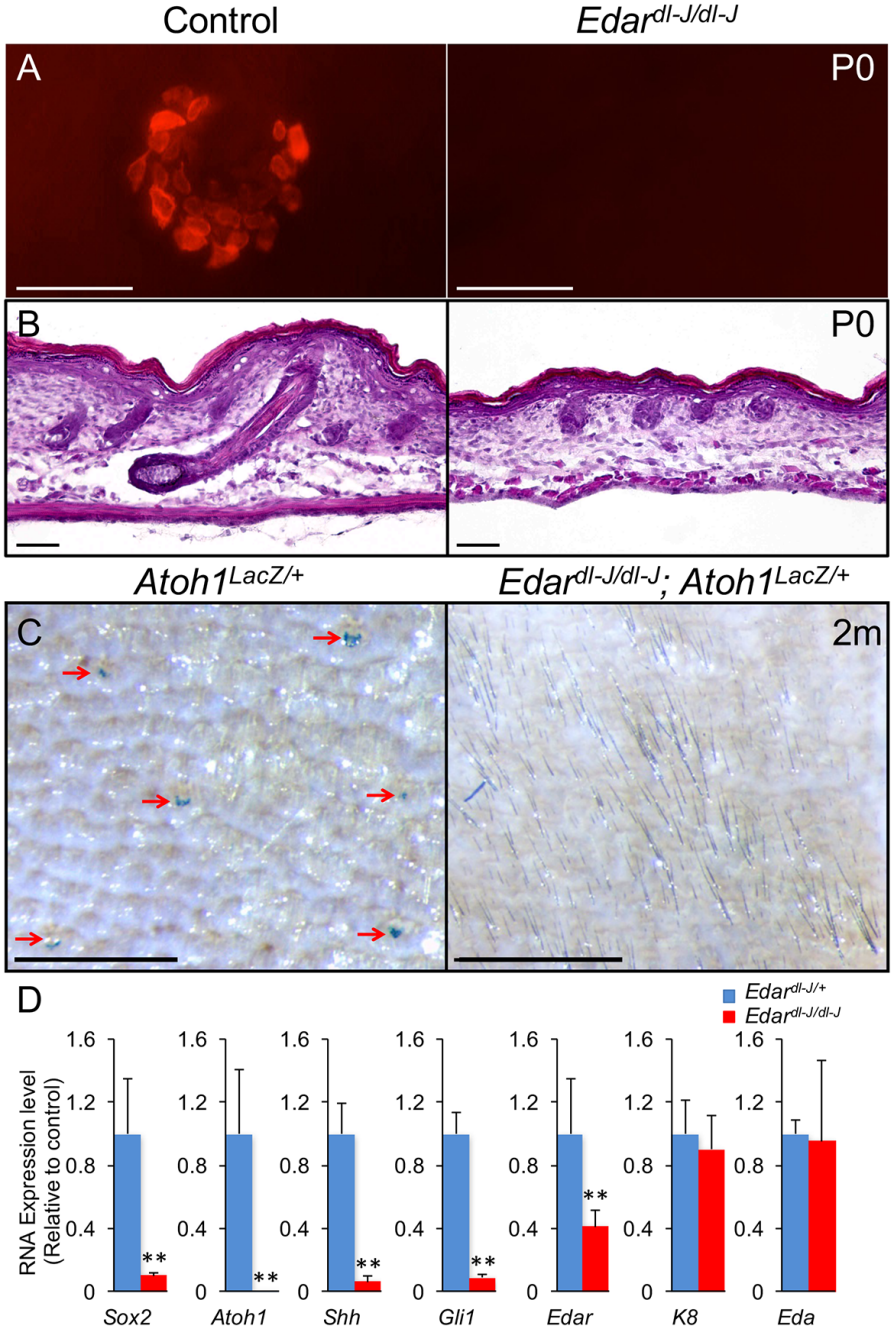
Edar is necessary for TD MC formation. (A) K8 whole mount staining in control (*Edar*^*dl-J/+*^) and *Edar*^*dl-J/dl-J*^ dorsal trunk skin at P0. (B) H&E staining in sections of control and *Edar*^*dl-J/dl-J*^ dorsal trunk skin at P0. (C) X-gal whole mount staining in *Atoh1*^*LacZ/+*^ and *Edar*^*dl-J/dl-J*^; *Atoh1*^*LacZ/+*^ dorsal trunk skin at 2 months old. (D) RT-PCR relative expression levels (mean ± SD) in control and *Edar*^*dl-J/dl-J*^ dorsal trunk skin at E15.5. **, *p*<0.01. Scale bars: A & B, 50 μm; C, 0.5 mm.

Dermal Wnt signaling is necessary for the earliest steps of epidermal patterning, including the upregulation of Edar expression in nascent hair placodes [22]. Edar is the membrane receptor for the TNF family protein Eda. MCs do not form in Eda mutant Tabby mice that have defective guard hair development [20]. We hypothesized that loss of Edar function would also disrupt MC development. To test this, we used point mutant *Edar*^*dl-J/dl-J*^ (downless) mice that lack guard hairs as their abortive primary hair germs fail to form follicles (n = 3, Fig 2B) [24]. No MCs were detected in postnatal day 0 (P0) dorsal trunk skin by whole mount K8 immunostaining (Fig 2A). We also failed to detect TD MCs in adult *Edar*^*dl-J/dl-J*^; *Atoh1*^*LacZ/+*^ mouse skin stained with X-gal (n = 3, Fig 2C) [25], demonstrating that TD MC formation is truly abrogated in the absence of Edar function, and not simply delayed. To examine the early molecular specification of TD MC, we examined gene expression in E15.5 *Edar*^*dl-J/dl-J*^ dorsal trunk skin (n = 3) using quantitative reverse transcription–PCR (RT-PCR). Relative to control skin, Edar mutant skin shows significantly reduced mRNA levels of the MC differentiation markers *Sox2* and *Atoh1* (Fig 2D), suggesting that MC specification itself is disrupted. A difference in K8 expression was not detected, likely due to the broad expression of K8 in the periderm of embryonic skin [26]. As Sox2 is also expressed in the forming dermal papilla of E15.5 primary hair germs [16], these results further suggest that dermal papilla differentiation is disrupted in *Edar*^*dl-J/dl-J*^ mouse skin. Together, these results strongly suggest that Edar signaling in the primary hair placode critically regulates TD MC formation and that Edar loss alone can explain the absence of TD MC development in the dermal β-catenin knockout skin.

### Shh Signaling Is Essential for TD MC Specification and Development

**Fig 3:**
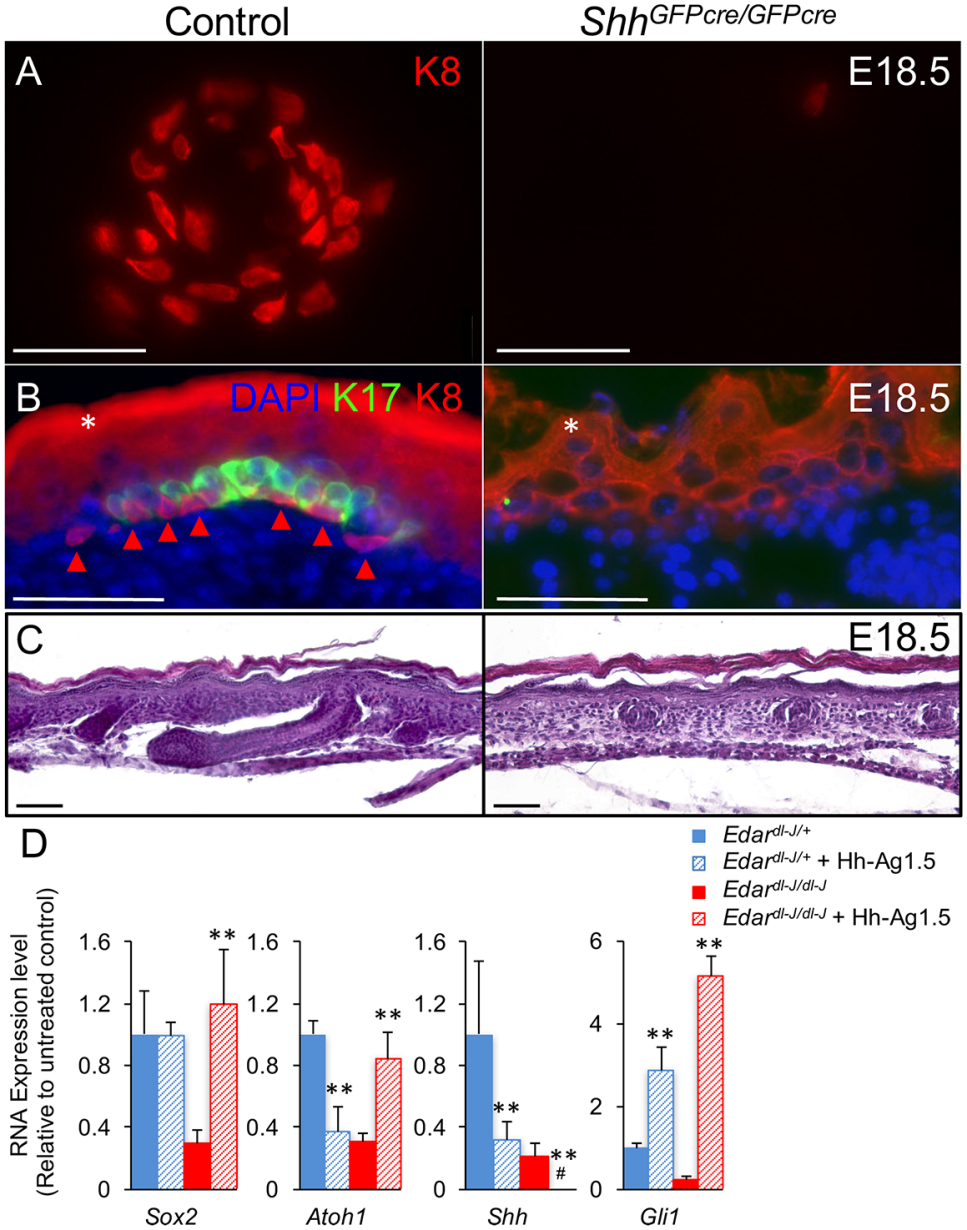
Shh is essential for TD MC development. (A) K8 whole mount staining in control (*Shh*^*GFPcre/+*^) and *Shh*^*GFPcre/GFPcre*^ skin at E18.5. (B) K8 and K17 staining in sections of control and *Shh*^*GFPcre/GFPcre*^ skin at E18.5. *, periderm and unspecific epidermal staining. Arrowhead, K8+ Merkel cell (C) H&E staining in sections of control and *Shh*^*GFPcre/GFPcre*^ skin at E18.5.(D) RT-PCR relative expression levels (mean ± SD) in skin from E13.5 *Edar*^*dl-J/+*^ and *Edar*^*dl-J/dl-J*^ embryos cultured for 2 days in the absence or presence of the Smo agonist Hh-Ag1.5. #, undetectable. **, *p*<0.01, compare to untreated. Scale bars, 50 μm.

There are a number of signaling molecules expressed downstream of Edar in nascent primary hair follicle that could potentially influence MC specification and development. Shh is a morphogen expressed by forming hair placodes [27], and *Edar*^*dl-J/dl-J*^ mice fail to express Shh in their abortive primary hair germs (Fig 2D) [24]. As nerve-derived Shh was recently shown to regulate TD stem cell renewal in adult mouse skin [6], we hypothesized that Shh production was the mechanism by which primary hair follicles regulate TD MC development. Embryos deficient for *Shh* survive to birth but die postnatally due to holoprosencephaly [28]. Shh-null mice have defective hair follicle development where hair germs form but do not elongate into follicles [29,30]. We generated *Shh*^*GFPcre/GFPcre*^ mouse embryos (n = 3) to assess TD MC formation in Shh null skin [31]. As expected, E18.5 *Shh*^*GFPcre/GFPcre*^ hair follicles were arrested as hair germs (Fig 3C). In E18.5 control epidermis (n = 3), K8+ TD MCs were observed in annular clusters around primary hair follicles (Fig 3A and 3B). In contrast, no TD structures were detected in Shh-null mouse embryos, and the epidermis contained very few scattered K8+ cells (Fig 3A and 3B), demonstrating that Shh signaling is essential for TD MC development.

Using the *Gli1*^*LacZ*^ reporter allele to visualize cells with active hedgehog signaling [32], we observed diffuse X-gal staining in E17.5 and P0 basal epidermis, dermis, and hair follicles (S1A and S1B Fig), demonstrating that there are hedgehog-responding cells throughout the epidermis of developing mouse skin. This finding is distinct from adult epidermis where *Gli1* expression is restricted to the mature touch dome [12,33]. No LacZ reporter expression was detected in P0 *Shh*^*GFPcre/GFPcre*^*; Gli1*^*LacZ/+*^ trunk skin (S1C Fig), suggesting that Shh is the primary Hh ligand regulating skin development.

To test if hedgehog-responding cells can serve as Merkel cell precursors, E15.5 *Gli1*^*CreER/+*^; *R26*^*YFP/+*^ embryos (n = 3) were treated with low-level tamoxifen (2mg to the gravid dam) to genetically label a fraction on Gli1+ cells at the time of MC specification [34]. Scattered labeled cells were visible in the primary hair follicles, dermis, and epidermis of P0 sectioned dorsal trunk skin (S2 Fig). GFP staining was found in 27.6% of K8+ Merkel cells (n = 333). This was comparable to GFP labeling in 24.3% of primary hair follicle epithelial cells (n = 1847), a structure derived from precursors that highly express hedgehog response genes at E15.5 [27]. These fate mapping results demonstrate that hedgehog-responding precursors give rise to TD MCs.

The requirement for Shh in TD MC formation suggests that Shh loss accounts for the MC defect in Edar mutant skin. To test whether Shh signaling was indeed the critical mechanism regulating MC formation downstream of Edar, we used the Smo agonist Hh-Ag1.5 to restore hedgehog signaling in embryonic Edar mutant skin during the window of MC specification. We cultured E13.5 *Edar*^*dl-J/dl-J*^ skin (n = 3), and after two days (E15.5), used RT-PCR to assess gene expression. In untreated cultures (n = 3), we observed reductions in *Sox2*, *Atoh1*, *Shh*, and *Gli1* relative to control skin (n = 3), similar to those seen in uncultured E15.5 Edar mutant skin (Figs 2D and 3D). Smo agonist treatment resulted in significant increases of *Gli1* transcription in both control and Edar mutant skin, confirming activation of hedgehog signaling. Smo agonist also resulted in a reduction of *Shh* expression in control skin, suggesting that some form of negative feedback downregulates *Shh* transcription. Similarly, Smo agonist further reduced *Shh* levels in Edar mutant skin. Interestingly, Smo agonist reduced *Atoh1* mRNA levels in control skin. This result is consistent with the observation that Shh signaling in neurons prevents Atoh1 degradation [35] and suggests that elevated Atoh1 protein levels in the setting of a Smo agonist can feedback to reduce *Atoh1* transcripts. Most importantly, Smo agonist rescued expression of the MC differentiation markers *Sox2* and *Atoh1* to normal levels in Edar mutant skin (Fig 3D). Together, these results suggest that Shh is both necessary and sufficient for MC formation in embryonic trunk skin and is the critical factor lost in Edar mutant skin.

### Shh from Early Primary Hair Follicles Is Required for TD Specification

**Fig 4:**
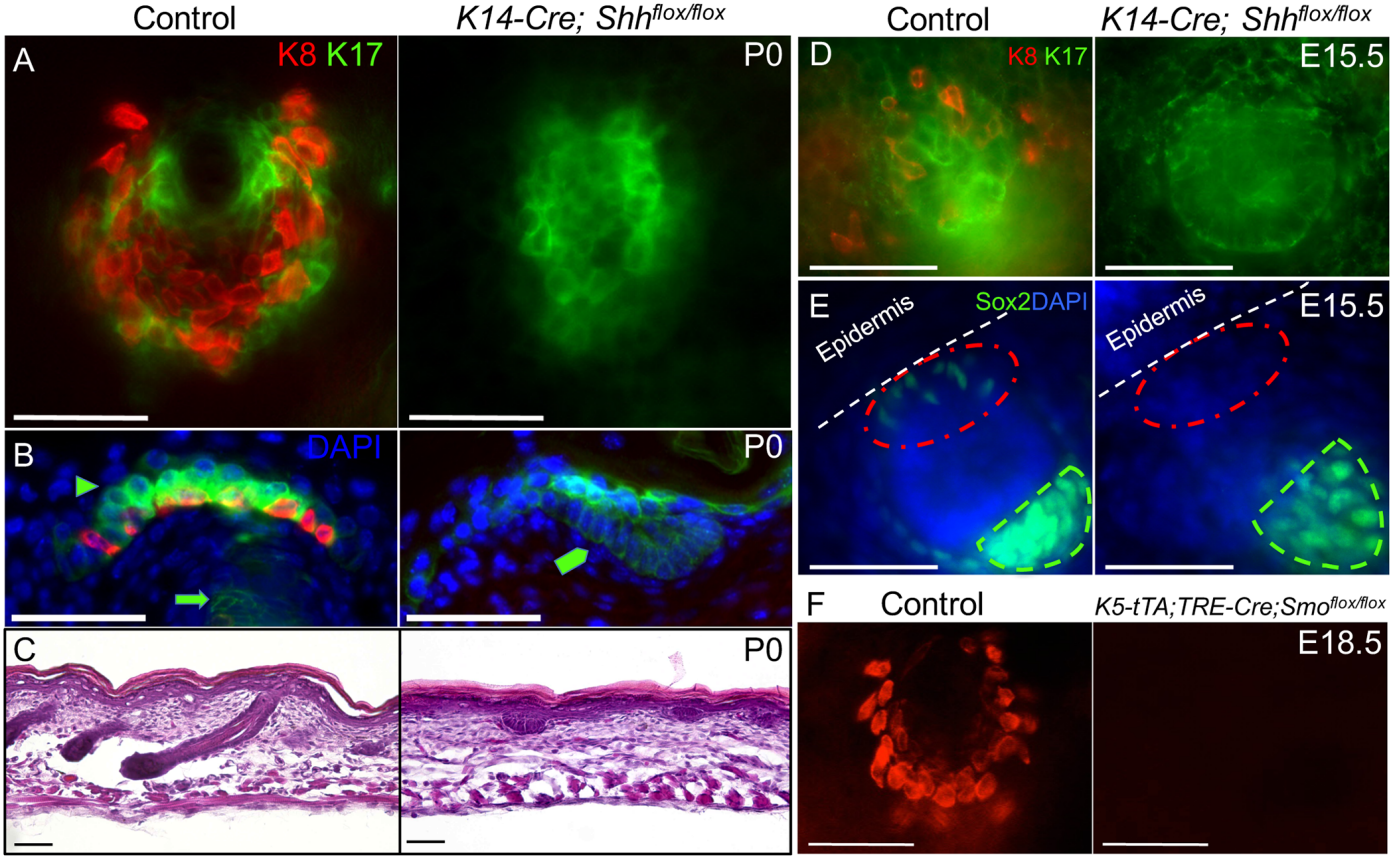
Intraepithelial Shh signaling is required for TD MC development. (A) K8 and K17 whole mount staining in control and *K14-Cre; Shh*^*flox/flox*^ skin at P0. (B) K8 and K17 section staining in control and *K14-Cre; Shh*^*flox/flox*^ skin at P0. Arrowhead, K17+ TD keratinocytes. Arrow, K17+ hair follicle. Pentagon, abortive K17+ hair germ. (C) H&E staining in sections of control and *K14-Cre; Shh*^*flox/flox*^ skin at P0. (D) K8 and K17 whole mount staining in control and *K14-Cre; Shh*^*flox/flox*^ skin at E15.5. (E) Confocal maximum projection oblique view of Sox2 whole mount staining in control and *K14-Cre; Shh*^*flox/flox*^ skin at E15.5. Red circle, epidermal surface over primary hair germ. Green outline, dermal papilla. (F) K8 whole mount staining in control and *K5-tTA; TRE-Cre; Smo*^*flox/flox*^ skin at E18.5. Scale bars, 50 μm.

In embryonic skin, Shh is initially expressed in hair follicle placodes and continues to be expressed in developing hair follicle bulbs [36]. However, Shh is also delivered to the skin by sensory nerves [33]. To test the requirement for follicle-produced Shh, we deleted *Shh* from the embryonic epidermis using *K14-Cre; Shh*^*flox/flox*^ mice [37,38]. Unlike control skin (n = 3), no TD MCs were detected in P0 mutant (n = 3) epidermis by immunostaining (Fig 4A and 4B), indicating that epidermal Shh is necessary for TD MC production. Similar to the Shh null mice, hair follicles failed to develop in *K14-Cre; Shh*^*flox/flox*^ skin. Abortive K17+ germ-like structures formed at sites of follicle induction but failed to elongate (Fig 4B, 4C and 4E). At the time of TD MC specification in E15.5 control skin (n = 2), we observed Sox2+ and K8+ cells in the epidermis above and adjacent to primary hair germs. In E15.5 *K14-Cre; Shh*^*flox/flox*^ skin (n = 2), neither Sox2 nor K8 staining was observed, suggesting that hair placode/germ-derived Shh is necessary for MC specification and that MC loss occurs prior to, and independent of, the follicle downgrowth defect in Shh mutant skin (Fig 4D and 4E). Sox2+ dermal papillae were observed under the abortive primary hair germs in E15.5 *K14-Cre; Shh*^*flox/flox*^ skin (Fig 4E), suggesting epidermal Shh is not necessary for dermal papilla specification. Next, we used *K5-tTA; TRE-Cre; Smo*^*flox/flox*^ mice in the absence of doxycycline to delete the obligate Shh signaling mediator Smo in the developing epidermis [39,40]. At E18.5, these mice (n = 2) completely lacked TD MCs that were readily detected by K8 immunostaining in control epidermis (n = 9, Fig 4F), demonstrating that the cells that require Shh signaling for TD MC development reside in the epidermis. Together, these results strongly suggest that intraepithelial Shh signaling from primary hair placodes/early hair germs to epidermal target cells is required for the specification and development of TD MCs.

### MCs Predominantly Form Outside the Hair Follicle Lineage

**Fig 5:**
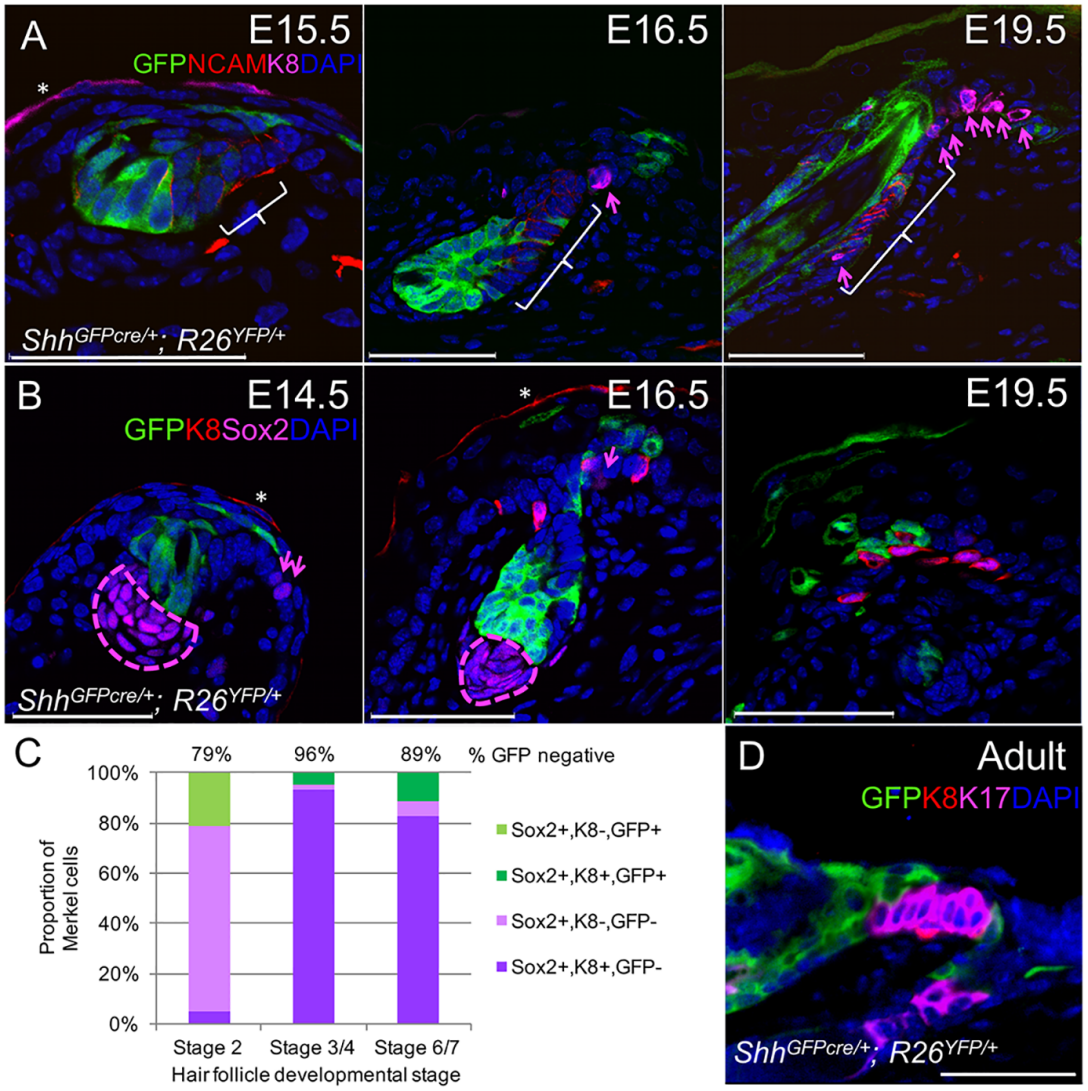
TD MCs predominantly arise from outside the hair follicle linage. (A) GFP, NCAM, and K8 section staining in *Shh*^*GFPcre/+*^*; R26*^*YFP/+*^ skin at E15.5, E16.5, and E19.5. Arrows, K8+ MCs. Bracket, NCAM+ caudal follicle with low YFP labeling. (B) GFP, K8, and Sox2 section staining in *Shh*^*GFPcre/+*^*; R26*^*YFP/+*^ skin at E14.5, E16.5, and E19.5. Arrows, Sox2+ K8- cells. Outline, dermal papilla. (C) Quantification of MCs with differential expression of Sox2, K8 and GFP in *Shh*^*GFPcre/+*^*; R26*^*YFP/+*^ skin sections at E14.5 (stage 2), E16.5 (stage 3/4), and E18.5/E19.5 (stage 6/7). (D) GFP, K8, and K17 section staining in an adult *Shh*^*GFPcre/+*^*; R26*^*YFP/+*^ skin. Scale bars, 50 μm. *, periderm staining.

Although K8+ cells seem to arise within forming primary hair follicles [20], our observations that Sox2+ and K8+ cells first appear in the epidermis above and adjacent to primary hair germs made us question whether TD MC progenitors develop within the hair placode or simply in close proximity to the hair follicle lineage. We used *Shh*^*GFPcre/+*^*; R26*^*YFP/+*^ mice to fate map the hair follicle lineage originating from the Shh-expressing hair placode [41]. To determine whether TD MCs arise from within the hair follicle lineage, we used immunostaining to assess co-labeling of Sox2+ and K8+ MCs with GFP staining in *Shh*^*GFPcre/+*^*; R26*^*YFP/+*^ skin at E14.5 (n = 3), E16.5 (n = 3), and E18.5/E19.5 (n = 3, Figs 5A, 5B, S3 and S4). In total (n = 189 MC), over 89% of MCs failed to stain with GFP, indicating that TD MCs primarily originate from extrafollicular cells. A breakdown of staining characteristics for MCs associated with hair follicles at different developmental stages [42] is shown in Fig 5C. A similar lack of GFP+ K8+ TD MCs was seen in adult *Shh*^*GFPcre/+*^*; R26*^*YFP/+*^ skin (Fig 5D), indicating that the stem cells maintaining TD MCs [13] also principally originate outside of the hair follicle lineage. Together, these results indicate that TD MCs predominantly arise from a developmental linage outside of primary hair placodes, although Shh+ ectodermal cells can give rise to a minor fraction of TD MCs.

The presence of Sox2+ K8- MCs that are more abundant in E14.5 skin versus later stages of development, and the complete absence of Sox2- K8+ cells (Figs 5C and S5A), suggests that Sox2 expression is an early event in MC specification and that K8 upregulation occurs later in MC differentiation. This finding is consistent with prior observations [16] and the fact that adult TD MC progenitors express very low levels of K8 and upregulate K8 expression upon differentiation to mature MCs [6,13].

In E14.5–16.5 skin, TD MCs were scattered in the epidermis adjacent to primary hair germs. In late embryonic and early postnatal skin (E18.5-P4), TD MCs were organized around the infundibulum of primary hair follicles, but were also found clustered on the upper caudal side of primary hair follicles, in a region known to express NCAM (Figs 5A, 5B, S3, S4, S5B, S5C) [20]. Interestingly, the MCs and NCAM+ keratinocytes found in *Shh*^*GFPcre/+*^*; R26*^*YFP/+*^ upper follicles infrequently expressed YFP (Figs 5A and S3). This observation suggests that the caudal side of the upper developing primary follicle does not form by downgrowth of the hair follicle placode but primarily by expansion of extraplacodal cells into the forming follicle. MCs do not persist in the upper region of primary follicles, as K8+ MCs are not detected within P13 guard hair follicles [6]. The purpose of the transient population of follicular MCs during late embryonic and early postnatal development is unclear.

### Fgf20 Is Dispensable in TD MC Development and Postnatal Maintenance

**Fig 6:**
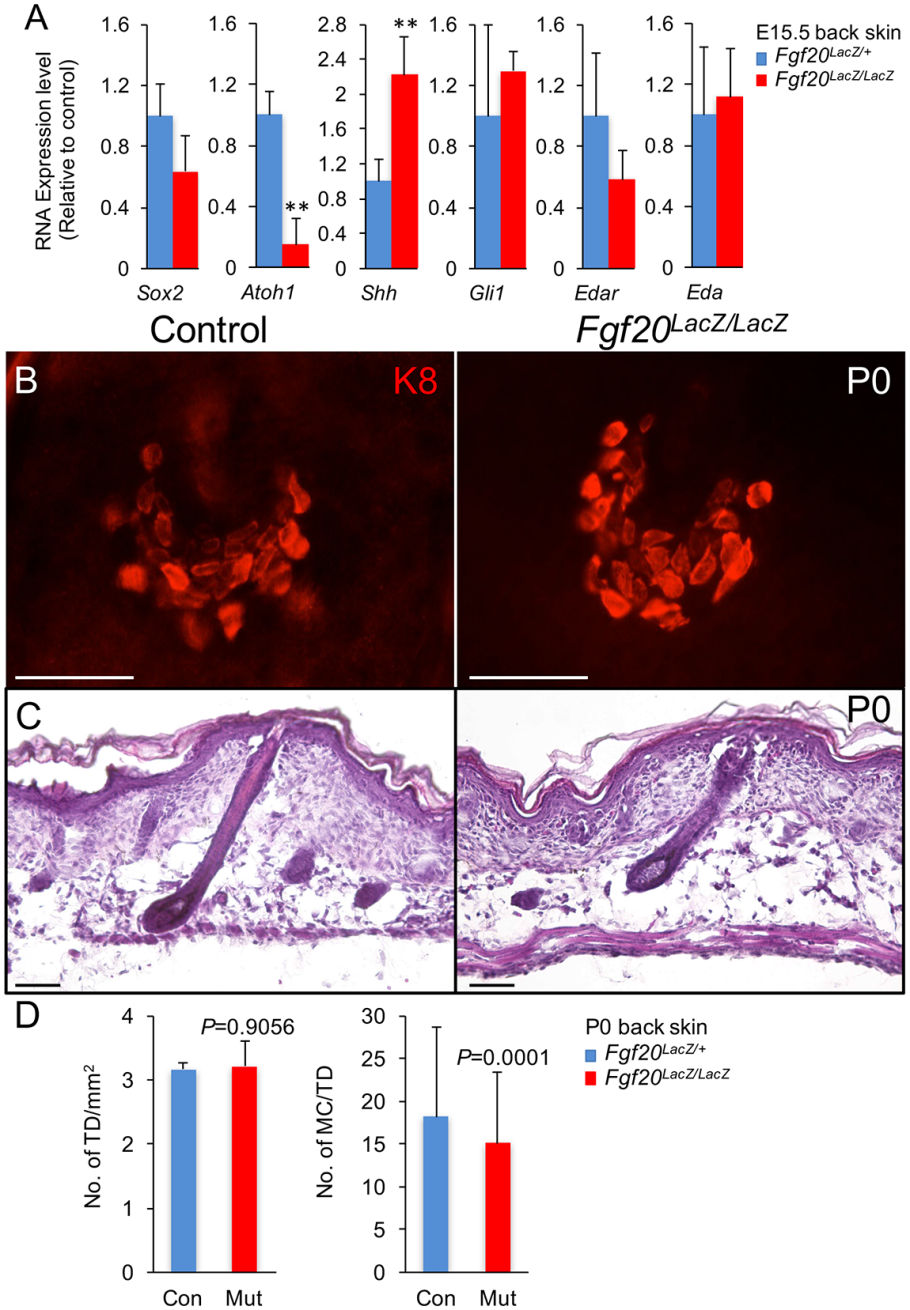
Fgf20 is not required for TD MC formation. (A) RT-PCR relative expression levels (mean ± SD) in control (*Fgf20*^*LacZ/+*^) and *Fgf20*^*LacZ/LacZ*^ dorsal trunk skin at E15.5. **, *p*<0.01. (B) K8 whole mount staining in control and *Fgf20*^*LacZ/LacZ*^ skin at P0. (C) H&E section staining of control and *Fgf20*^*LacZ/LacZ*^ skin at P0. (D) Quantification of TD density per mm^2^ and MC number per TD in control and *Fgf20*^*LacZ/LacZ*^ dorsal trunk skin at P0. Scale bars, 50 μm.

We used mice with dermal β-catenin deletion, *Edar* mutation, *Shh* mutation, epidermal deletion of *Shh*, and epidermal deletion of *Smo* to show that paracrine Shh signaling within the epidermis, downstream of Wnt and Eda signaling, is essential for TD MC development. However, in all of these mice, there is either a global defect in hair follicle patterning and development, a developmental defect in all hair follicles, or a defect in primary hair follicles. To separate Shh signaling from normal guard hair development, we used Fgf20-null (*Fgf20*^*LacZ/LacZ*^) mice [43]. Like Shh, Fgf20 is expressed by hair placodes and is regulated by Wnt/β-catenin and Eda/Edar signaling. In Fgf20 mutant skin, there is a defect in the formation of hair follicle-associated dermal papillae and a failure in the downgrowth of primary hair germs [43]. Although the abortive primary hair germ phenotype of Fgf20 mutant skin resembles that of Eda and Edar mutant skin, Shh expression is preserved in the defective primary hair follicles [43]. We used RT-PCR to confirm that Shh is expressed in E15.5 *Fgf20*^*LacZ/LacZ*^ skin (n = 3) and found *Shh* levels elevated compared with those in control skin (n = 3, Fig 6A). A small, nonsignificant increase in *Gli1* mRNA levels was also observed. In P0 Fgf20 mutant skin (n = 3), we occasionally observed larger follicles that were comparable to guard follicles in control skin (n = 3, Fig 6B), suggesting that after an initial arrest, primary follicle downgrowth can occur. Nonetheless, the primary follicles were not normal, as guard hairs were absent in the coats of juvenile and adult *Fgf20*^*LacZ/LacZ*^ mice [43]. In E15.5 *Fgf20*^*LacZ/LacZ*^ skin, there were decreased levels of *Edar*, *Sox2*, and *Atoh1* expression, although only the *Atoh1* reduction reached statistical significance (Fig 6A), and a *Sox2* reduction was expected based on the dermal papilla defect in this mouse. Despite the reduction in MC factors at the time of MC specification, the pattern and distribution of K8-immunostained TD MCs was normal in P0 *Fgf20*^*LacZ/LacZ*^ skin (n = 4 mice, 752 TD, 11,393 MC, Fig 6B and 6D). We did observe a 17% reduction in the mean number of MC per TD relative to control epidermis (n = 2 mice, 348 TD, 6,338 MC, Fig 6B and 6D). Normal-appearing TDs persisted into adulthood with a normal TD density and a continued reduction (14%) in MC/TD observed in adult (P50-P103) *Fgf20*^*LacZ/LacZ*^ mice (n = 3 mice, 131 TD, 1685 MC) relative to control (n = 3 mice, 136 TD, 1979 MC, S6 Fig). These results demonstrate that Fgf20 is required for normal guard hair follicle development but is not necessary for TD MC specification; our results also illustrate that when Shh expression is preserved, even abnormal primary hair germs are capable of supporting TD MC development.

## Discussion

**Fig 7:**
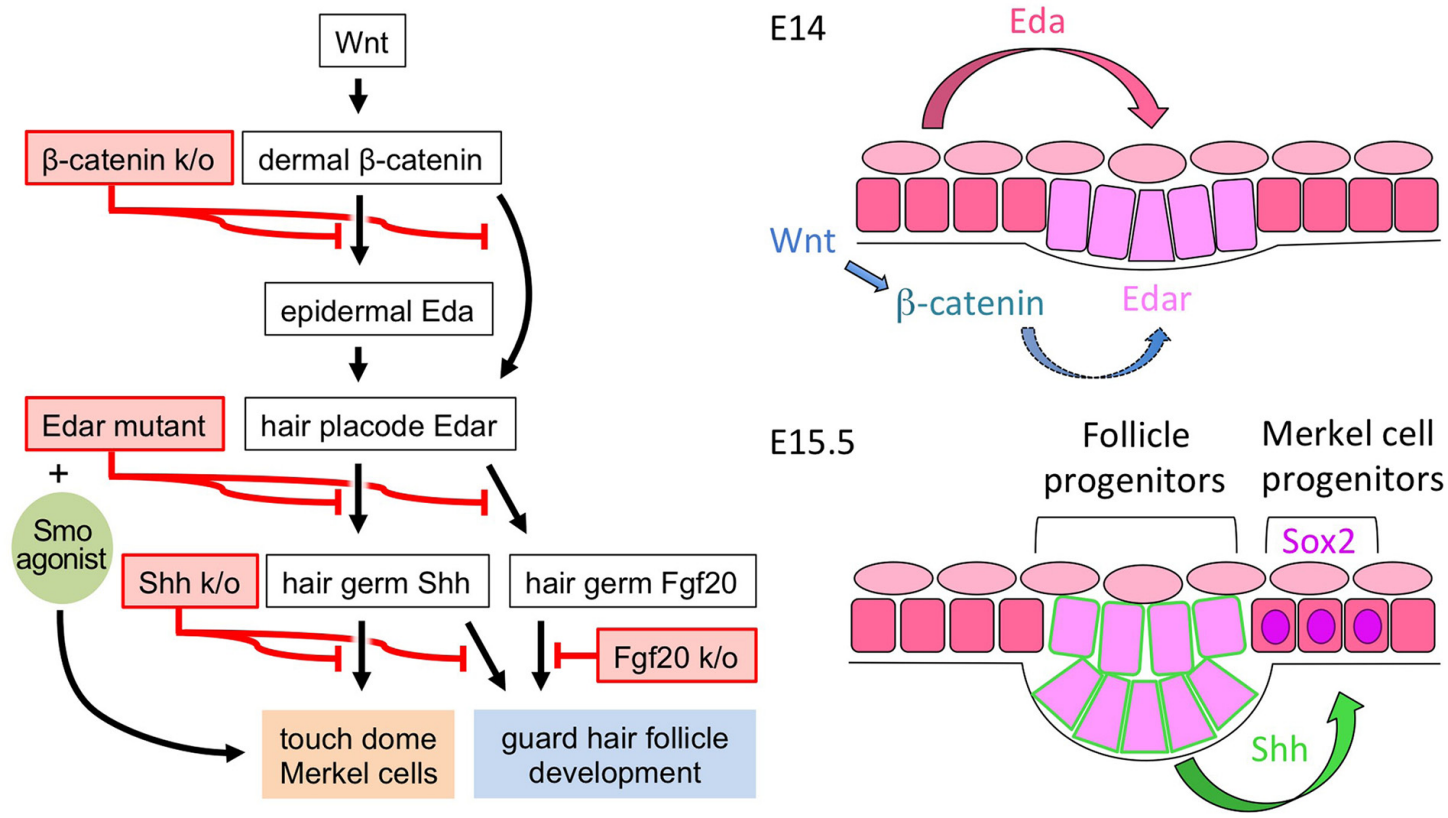
Proposed model of the developmental signaling requirements for TD MC production. (left) Model of the signaling cascade that regulates the development of TD MCs and their adjacent guard hair follicle, along with experimental interventions that disrupted or rescued the developmental processes. (right) Graphical representation of the sequential signaling mechanisms that drive TD MC specification in embryonic mouse skin. TD MC specification requires mesenchymal Wnt signaling which allows Eda signaling to Edar in the developing primary hair placodes and subsequent Shh signaling from the primary hair placodes/germs to adjacent ectoderm containing the TD anlages. Square cells, surface ectoderm. Trapezoidal cells, hair placode/germ cells. Oval cells, periderm.

Vertebrate skin provides barrier, mechanical, defensive, communicative, sensory, metabolic, and homeostatic functions. This diversity of function is achieved, in part, by specialization of ectodermal appendages that form through a series of mesenchymal-epithelial interactions in the embryo. By regulating common inductive events with similar yet distinct sets of morphogens, great diversity of structure and function is achieved [7]. Although some types of ectodermal appendages are specific to mammals (hair follicles, mammary, and sweat glands), structures that enhance sensation of the outside world are common adaptations across all classes of vertebrates. We have used multiple genetically modified mouse models to elucidate for the first time the developmental signaling cascade required for the formation of sensory TDs in embryonic skin. Only recently was the TD discovered to be a distinct skin lineage, maintained by its own resident stem cells. Here, we find that the mechanism for establishing the TD lineage within the developing ectoderm requires many of the morphogens that drive formation of ectodermal appendages. We discovered that TD MC specification requires Wnt-dependent mesenchymal signals to establish reciprocal signaling within the developing ectoderm, including Eda signaling to primary hair placodes, and subsequent Shh signaling from primary follicles to extrafollicular MC progenitors (Fig 7). Our identification of primary follicle Shh as a critical regulator of MC specification is consistent with TD development being spatially and temporally associated with the first wave of hair follicle induction in embryonic trunk skin, whereas our fate mapping results confirm that the TD lineage is separate from adjacent hair follicles. Together, our findings identify the TD as a distinct ectodermal touch receptor whose development is critically regulated by Wnt, Eda/Edar, and Shh signaling.

Our results support a model of TD MC development in which MC specification is tied to primary hair follicle patterning and morphogenesis up to the point of Shh production by the early follicle and can diverge from follicle development with signals such as Fgf20 that act in parallel or downstream to Shh in the hair follicle. Similarities in the developmental signaling pathways between the TD and primary hair follicles include the importance of mesenchymal Wnt signaling and ectodermal Eda/Edar signaling. Accordingly, there is no Shh expression by primary hair placodes in *En1*^*Cre/+*^*; β-catenin*^*flox/Δ*^ skin, *Edar*^*dl-J/dl-J*^ skin, or Eda mutant Tabby skin [44], and TD MCs fail to form in all these mice. Although Shh signaling is critical in the development of both hair follicles and TDs, the role of Shh appears different for each structure. In the hair follicle, Shh signaling is dispensable for placode induction but is needed for the subsequent growth of the follicle. In the TD, loss of Shh signaling by either deleting Shh production or removing epidermal Smo results in complete loss of MC specification and development—a phenotype that is evident even before changes are seen in the primary hair germ. Another contrasting feature is that a hedgehog signaling agonist was sufficient to rescue MC differentiation in Edar mutant skin, whereas transgenic expression of *Shh* in Eda mutant Tabby mice failed to restore primary hair follicle development [45]. Downstream of Eda/Edar signaling, Fgf20 expression in the developing hair placode is needed for proper dermal papilla and guard hair formation, while Shh is expressed and TD MCs are able to form. Thus, although TDs and hair follicles share early developmental requirements for Wnt and Eda signaling, they arise from distinct ectodermal compartments, are maintained as distinct lineages, and differ in their specific requirements for Shh and Fgf20.

The coats of Fgf20 mutant mice lack guard hairs, and yet typical-appearing TDs were found at normal density and only a slightly reduced average number of MCs per TD. Interestingly, TD MCs are maintained in adult *hairless* mice, where hair follicles develop normally but undergo cystic degeneration after the first month of life [6], demonstrating that once established, the TD lineage can persist without an affiliated guard follicle. Thus, the primary hair follicle is a necessary source of Shh for TD MC formation in embryonic skin but is dispensable in postnatal MC maintenance. In postnatal mouse skin, maintenance of TD stem cells requires Shh signaling from sensory neurons that innervate MCs. However, TDs are specified at a time prior to epidermal innervation [46], and embryonic deletion of *Shh* from sensory neurons had no impact on TD MC formation [6]. It is noteworthy that Shh critically regulates different TD functions during development and in postnatal skin. In the embryo, hair follicle Shh is required to establish the MC lineage. Postnatally, loss of neural Shh blocks the maintenance of TD stem cells, but MC differentiation continues until the progenitor pool is exhausted [8]. This is analogous to the developing telencephalon, where Shh from the prechordal mesoderm and ventral forebrain is a critical morphogen for neural patterning and development [47], however postnatal neural stem cells are maintained by Shh from local niche neurons [48,49]. Shh signaling often shows pleiotropic effects within a given organ system with roles in patterning, specification, and proliferation during development and later functions in stem cell regulation, tissue regeneration, and cancer formation. Our findings and the observations from the central nervous system suggest that altering the source of ligand is an important contextual component influencing the function of Shh signaling within a tissue.

The formation of TDs adjacent to primary hair follicles requires Shh signaling from the nascent follicles. Secondary and tertiary hair follicles also express Shh during their formation, and yet TDs do not form in association with induction of those hair follicles. Moreover, based on the broad expression of Gli1 within the developing epidermis, many embryonic epidermal cells receive Shh signaling. Thus, even though a hedgehog signaling agonist was sufficient to rescue MC differentiation in Edar mutant skin, Shh signaling alone is not sufficient to induce TD MC specification in any and all developing ectoderm. Further experimentation will be necessary to identify the factors that establish the temporal and spatial competency of developing epidermis to respond to Shh signaling with MC specification. However, Polycomb repressive complex 2 (PRC2) activity appears to be important in preventing MC specification around secondary and tertiary hair follicles (personal communication, E. Ezhkova). Notch signaling may also play a role in limiting MC specification, as ectopic expression of Atoh1 is able to induce MC production in some epithelial compartments of the skin, and impeding Notch signaling facilitates this process [50].

Fate mapping showed that MCs arise predominantly from ectoderm outside the forming hair follicle. This is consistent with our observations that the first MCs appeared as Sox2+ cells in the E14.5/15.5 epidermis above and adjacent to forming primary hair germs. We confirmed that late embryonic and early postnatal guard hair follicles contain a discrete population of MCs, however these cells also tend to arise from outside the hair follicle lineage. Although the purpose of the MCs in the upper regions of primary hair follicles during development is unclear, it has been proposed that the NCAM expressed on keratinocytes around these MCs may facilitate MC innervation [20]. Coincidently, the early postnatal window corresponds to a period when TD MC innervation undergoes pruning and maturation [46][51], and the onset of perineural influence on MC maintenance [6]. Currently, it is not known whether the MCs in the follicle during this period eventually die, change their fate, or migrate to TDs in the epidermis.

We have determined that intraepithelial Shh signaling from the developing hair follicle to MC precursors is a critical factor in TD MC production. However, the precise location of the target cells for Shh remain undefined. Because the Hh response gene *Gli1* is broadly expressed in the developing epidermis, it cannot be used to identify the specific K5+ Shh-responding cells required for TD MC development. Just as adult TD stem cells are Gli1+ [6], our Gli1 fate mapping experiments in the embryo show that TD MC progenitors are a direct target of Shh signaling. Because MC development within the TD anlage is dependent on Shh signaling from developing hair follicles, it is reasonable to assume the TD anlage forms in proximity to primary hair placodes. Moreover, the appearance of early MCs in the epidermis around primary hair germs and the observation that a minor portion of TD cells originate from the hair placode lineage suggest that the TD anlage is likely the ectoderm immediately adjacent to, and slightly overlapping, the primary hair placode. It is uncertain whether the TD forms from its own placode and associated mesenchymal condensate; however, this is unlikely, as no such structures have been observed. It is more likely that the TD is induced in the adjacent ectoderm by inchoate primary hair follicles or by the same signals that induce the follicles. Nonetheless, as an independent epidermal lineage requiring mesenchymal induction during development, and being absent in mouse models of ectodermal dysplasia (Eda and Edar mutant mice), TDs can be considered ectodermal appendages, or at least accessory structures to ectodermal appendages.

This work elucidates the developmental signaling requirements for the sensory TD and illustrates the commonalities and contrasts that exist between TD development and development of the closely associated guard hair follicle. These results further our functional understanding of skin patterning and development, the repertoire of ectodermal appendage formation, and how specialized sensory structures form prior to interfacing with the sensory nervous system. Intriguingly, along with prior observations, these findings indicate that the distinct functions of Shh signaling in TD development and maintenance correspond to changes in the source of the Shh ligand required for the varied effects.

## Materials and Methods

### Animals

Mice were housed and bred on an outcrossed Swiss Webster background in a pathogen-free facility at the National Cancer Institute (NCI), Bethesda, MD. Genotyping of mice was performed by allele-specific PCR on DNA extracted from tail tissue. All experiments were performed in accordance with institutional guidelines according to IACUC-approved protocols. *En1*^*Cre/+*^*; β-catenin*^*flox/Δ*^ and control samples were provided by Dr. Radhika P. Atit. Some *Shh*^*GFPcre/+*^; *R26*^*YFP/+*^ samples were provided by Dr. Sunny Y. Wong. *Edar*^*dl-J/+*^, *Atoh1*^*LacZ/+*^, *Shh*^*GFPcre/+*^, *Gli1*^*LacZ/+*^, *Gli1*^*CreER/+*^, *K14-Cre*, *Shh*^*flox/+*^, *K5-tTA*, *TRE-cre*, *Smo*^*flox/+*^, *R26*^*YFP/+*^, *R26*^*LacZ/+*^, and *Fgf20*^*LacZ/+*^ mice were described previously as cited in the text. For all embryonic and neonatal observations, littermate animals with a wildtype copy of the targeted allele and/or lacking cre recombinase activity were used as controls.

### Animal Treatments

*K5-tTA; TRE-cre; Smo*^*flox/flox*^ mice were bred and maintained on a standard rodent diet without doxycycline during embryonic development. Tamoxifen (Sigma) was dissolved in corn oil (20mg/ml) and administered (2mg intraperitoneal injection per gravid mouse) to induce CreER.

### Tissue Processing

Skin was fixed in 4% paraformaldehyde for 15 minutes (for X-gal staining) or overnight (for immunostaining). Tissue was whole mount-stained or cryoprotected overnight in 30% sucrose, embedded in OCT, and frozen; 12-μm sections were obtained.

### Immunofluorescent Staining

Standard and whole mount immunostaining procedures were performed. Tissue sections on glass slides were fixed in 4% paraformaldehyde for 15 minutes before incubation in 10% serum in 0.1% PBT (0.1% Triton X-100 in PBS) for 1 hour and then in primary antibody (in 5% serum/0.1% PBT) overnight at 4°C. The primary antibodies used were: rat anti-K8 (1:50, University of Iowa), rabbit anti-K17 (1:200, Epitomics), chicken anti-GFP (1:1000, Abcam), rabbit anti-GFP (1:500, Abcam), rabbit anti-Sox2 (1:500, Stemgent), and rabbit anti-NCAM (1:500, Millipore). Alexa Fluor-conjugated secondary antibodies (1:2000, Invitrogen) were used to detect the signals. Whole mount immunostaining followed the online protocol as described [52]. Concomitant staining of littermate control tissue and control staining where the primary antibody was omitted were used to confirm the specificity of experimental staining. Confocal images were acquired with the Zeiss LSM 710 Confocal system (Carl Zeiss Inc, Thornwood, NY).

The Troma-1/K8 antibody developed by Dr. Philippe Brulet and Dr. Rolf Kemler was obtained from the Developmental Studies Hybridoma Bank, developed under the auspices of the National Institute of Child Health and Human Development and maintained by The University of Iowa, Department of Biology, Iowa City, IA 52242.

### Quantification of Merkel Cells

Merkel cells per touch dome and touch dome per mm^2^ numbers were assessed by direct visualization of immunofluorescently stained K8+ Merkel cells in whole mount dorsal trunk skin from experimental and control animals. Counting was performed by a blinded observer. In tissue sections, Merkel cells were visualized by K8 and/or Sox2 staining and were scored for co-staining with GFP. Reporter recombination in primary hair follicles was quantified by counting total GFP staining cells and dividing by total number of DAPI stained nuclei within a preselected region of each follicle.

### Detection of Relative RNA Expression in Embryonic Skin

Total RNA from embryonic trunk skin was purified using RNeasy Micro Kit (Qiagen) and reverse-transcribed into cDNA following the manufacturer’s manual (Invitrogen #11752). Then quantitative RT-PCR, with actin as control, using SYBR Green was performed to detect RNA expression. Data are presented as means ± SD. The sequences of PCR primers are shown in S1 Table.

### Mouse Embryonic Skin Culture

Embryonic skin culture was performed as described [53]. E13.5 embryos were collected, each embryonic mouse was cut through the sagittal midline and eviscerated, half was cultured in media (DMEM +10% FBS +1% penicillin/streptomycin) as a control group, and half was cultured in media with 25 nM Smo agonist Hh-Ag1.5 (Xcess Biosciences Inc.) as the treated group. Media was changed the next day, and skin was harvested 2 days after culture, followed immediately by RNA isolation.

### Statistical Analyses

Population data sets are shown as the mean values, and error bars represent SD. For comparisons between sets, a two-tailed t-test was applied.

## Supporting Information

S1 Fig Shh is the signaling ligand driving Gli1 expression in developing skin. (A, B) X-gal section staining in *Gli1*^*LacZ/+*^ dorsal trunk skin at E17.5 and P0. (C) X-gal whole mount staining viewed from dermis side in *Gli1*^*LacZ/+*^ and *Shh*^*GFPcre/GFPcre*^*; Gli1*^*LacZ/+*^ skin at P0.
(TIF)

S2 Fig Gli1+ hedgehog-responding precursors give rise to touch dome MCs. (A) Schematic of the experimental design to fate map cells expressing Gli1 at E15.5. (B) GFP and K8 section staining in *Gli1*^*CreER/+*^*; R26*^*YFP/+*^ skin at P0. Arrow, K8+ GFP+ Merkel cell. Outline, basement membrane. (C, D) Individual florescent channels for inset in B. (E) Quantification of GFP+ labeled cells in the primary follicle epithelium and Merkel cells in P0 dorsal trunk skin.
(TIF)

S3 Fig Individual florescent channels for Fig 5A. (TIF)

S4 Fig Individual florescent channels for Fig 5B and 5D. (A) Individual florescent channels for Fig 5B (B) Individual florescent channels for Fig 5D.
(TIF)

S5 Fig Sox2+ K8- MCs form in the touch dome and caudal upper follicle. (A) En face image of Sox2 and K8 whole mount staining in wildtype P0 and P4 touch domes. (B) X-gal section staining in *Atoh1*^*LacZ/+*^ skin at P0 and K8 and K17 section staining in wildtype skin at P0. (C) Confocal maximum projection oblique view of Sox2 and K8 whole mount staining in wildtype skin at P0. Arrows, touch dome in epidermis. Arrowheads, MCs in upper hair follicle. Scale bars, 50 μm.
(TIF)

S6 Fig Fgf20 is dispensable in maintaining TD MCs in adult skin. (A) K8 whole mount staining in control (*Fgf20*^*LacZ/+*^) and *Fgf20*^*LacZ/LacZ*^ skin at P50. Scale bar, 50 μm. (B) Quantification of TD density per mm^2^ and MC number per TD in control and *Fgf20*^*LacZ/LacZ*^ dorsal trunk skin of adult (P50 –P103) mice.
(TIF)

S1 Table Primers used for quantitative RT-PCR. (PDF)
