# Supplementary figures and images for: A Cascade of Wnt, Eda, and Shh Signaling Is Essential for Touch Dome Merkel Cell Development
Source: PLoS Genet. 2016 Jul 14;12(7):e1006150. doi: 10.1371/journal.pgen.1006150 (PMC4944988; doi:10.1371/journal.pgen.1006150)

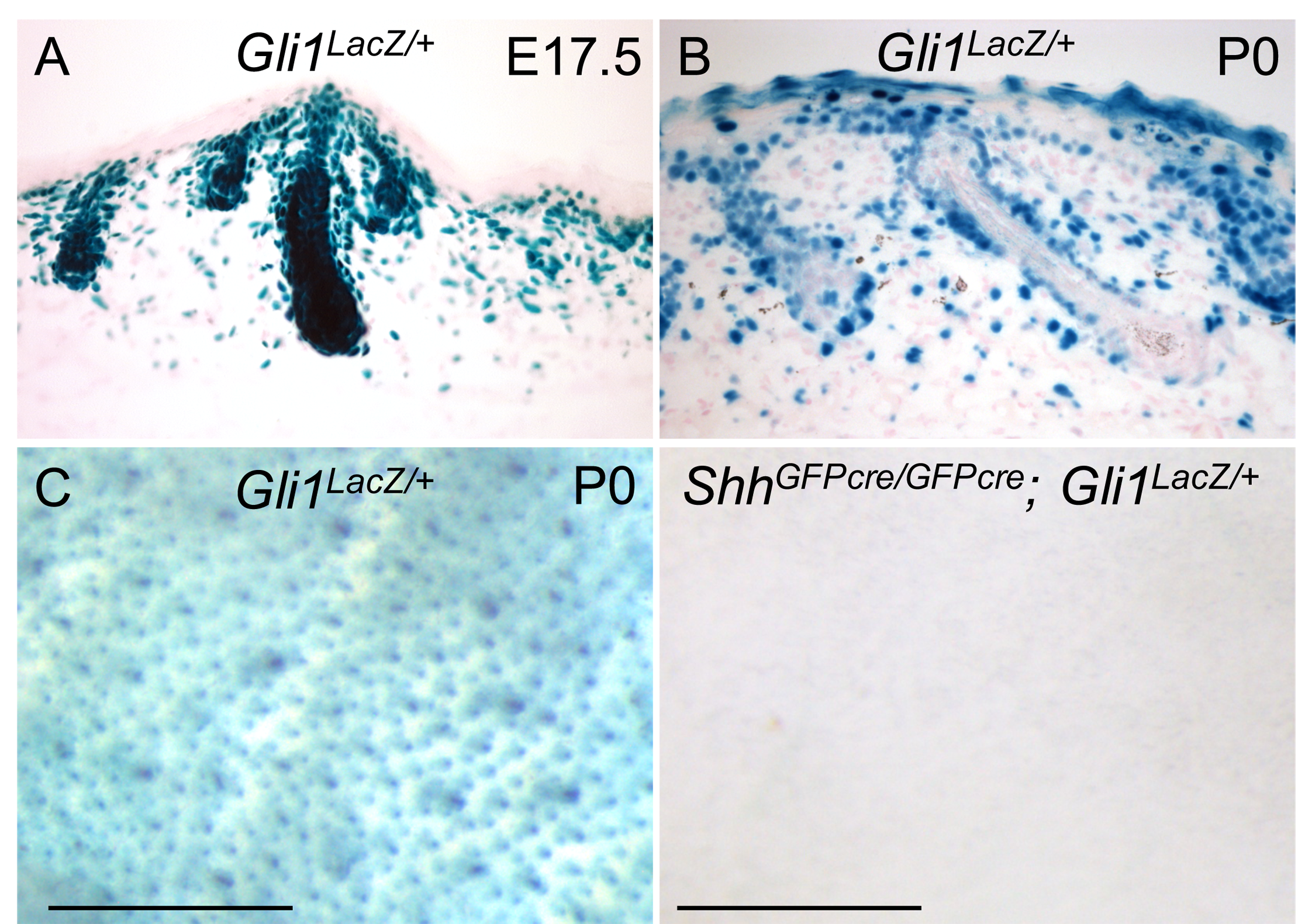

Supplement: S1 Fig — (A, B) X-gal section staining in Gli1LacZ/+ dorsal trunk skin at E17.5 and P0. (C) X-gal whole mount staining viewed from dermis side in Gli1LacZ/+ and ShhGFPcre/GFPcre; Gli1LacZ/+ skin at P0. (TIF) [file pgen.1006150.s001.tif]

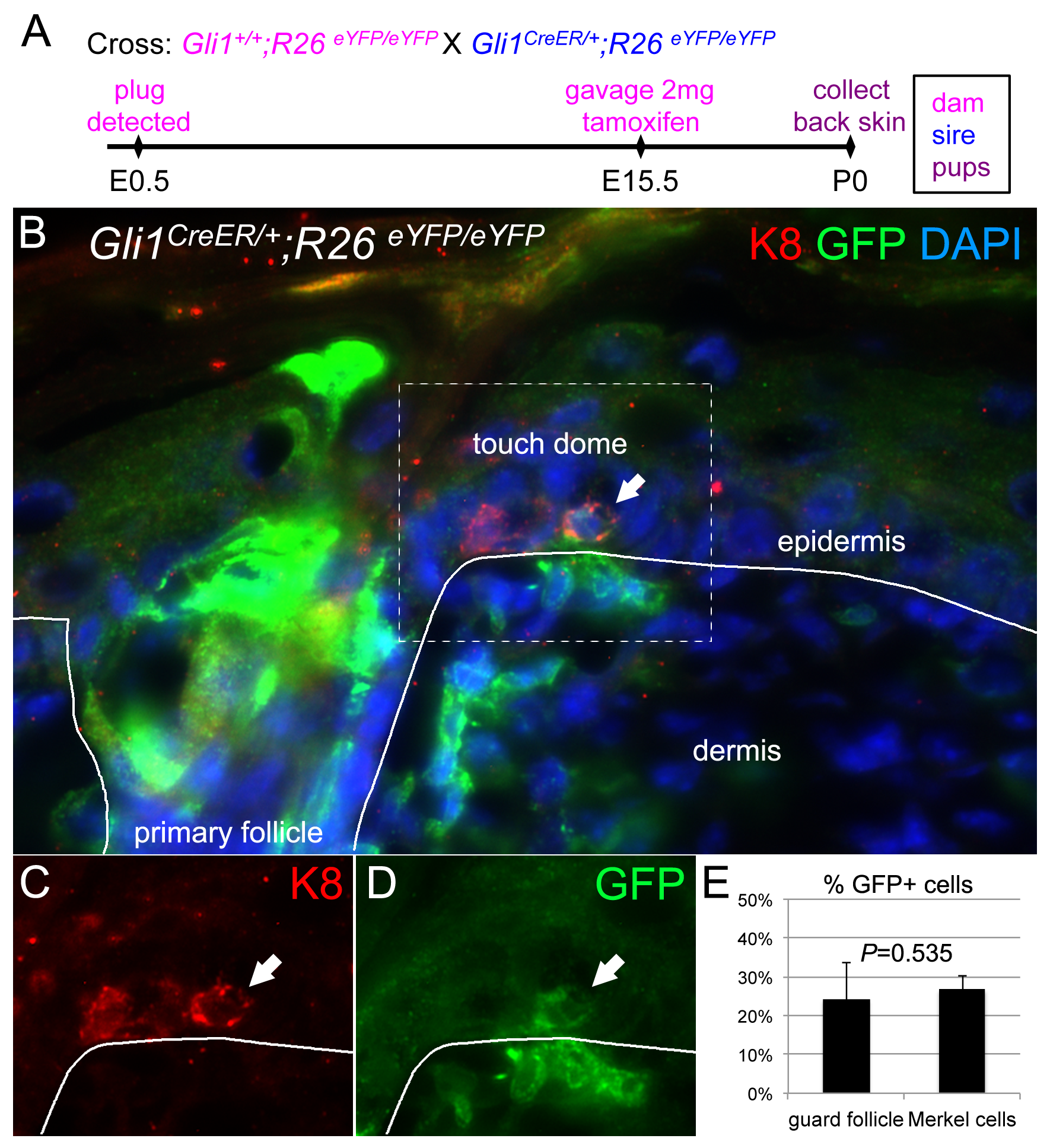

Supplement: S2 Fig — (A) Schematic of the experimental design to fate map cells expressing Gli1 at E15.5. (B) GFP and K8 section staining in Gli1CreER/+; R26YFP/+ skin at P0. Arrow, K8+ GFP+ Merkel cell. Outline, basement membrane. (C, D) Individual florescent channels for inset in B. (E) Quantification of GFP+ labeled cells in the primary follicle epithelium and Merkel cells in P0 dorsal trunk skin. (TIF) [file pgen.1006150.s002.tif]

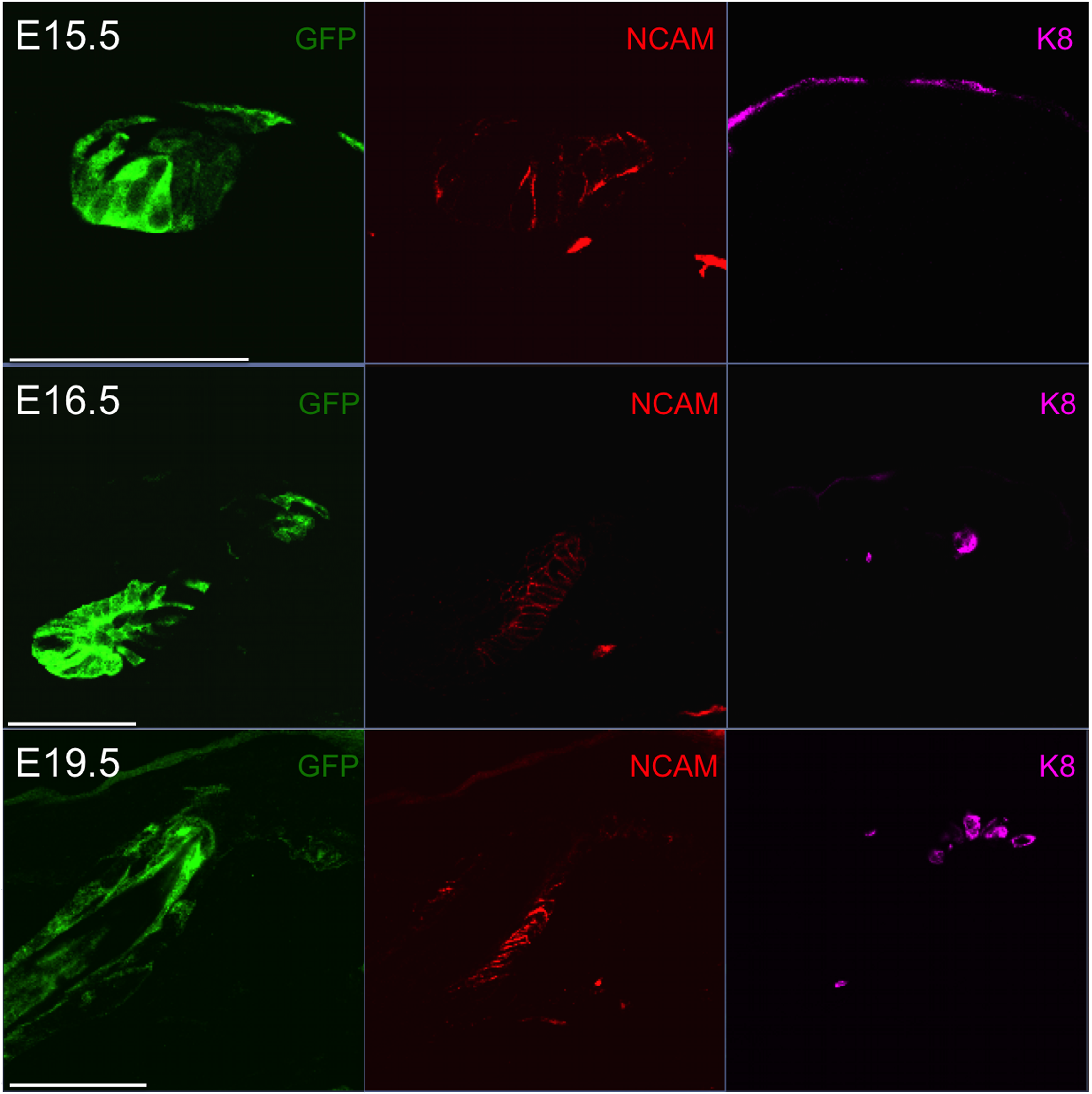

Supplement: S3 Fig — (TIF) [file pgen.1006150.s003.tif]

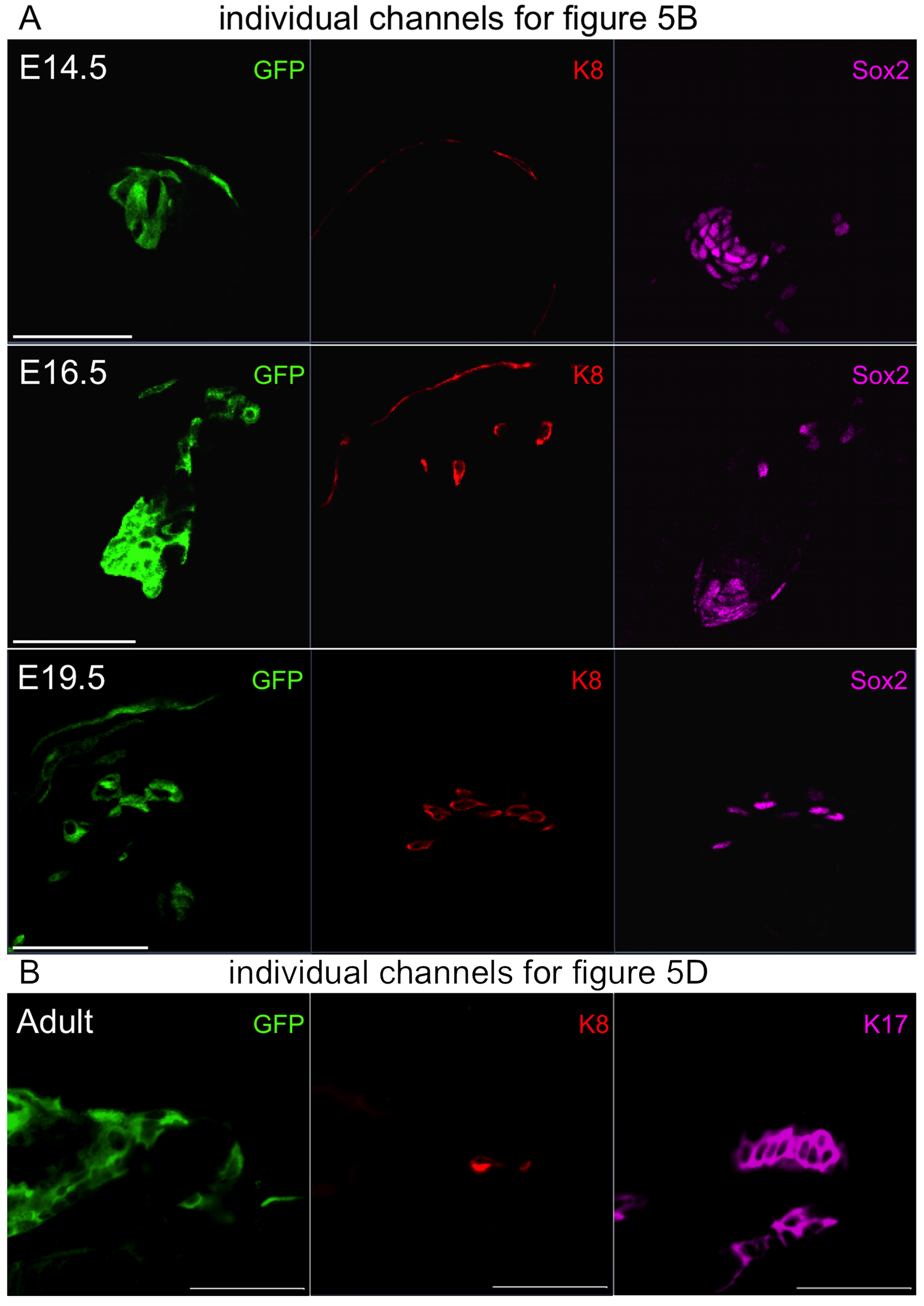

Supplement: S4 Fig — (A) Individual florescent channels for Fig 5B (B) Individual florescent channels for Fig 5D. (TIF) [file pgen.1006150.s004.tif]

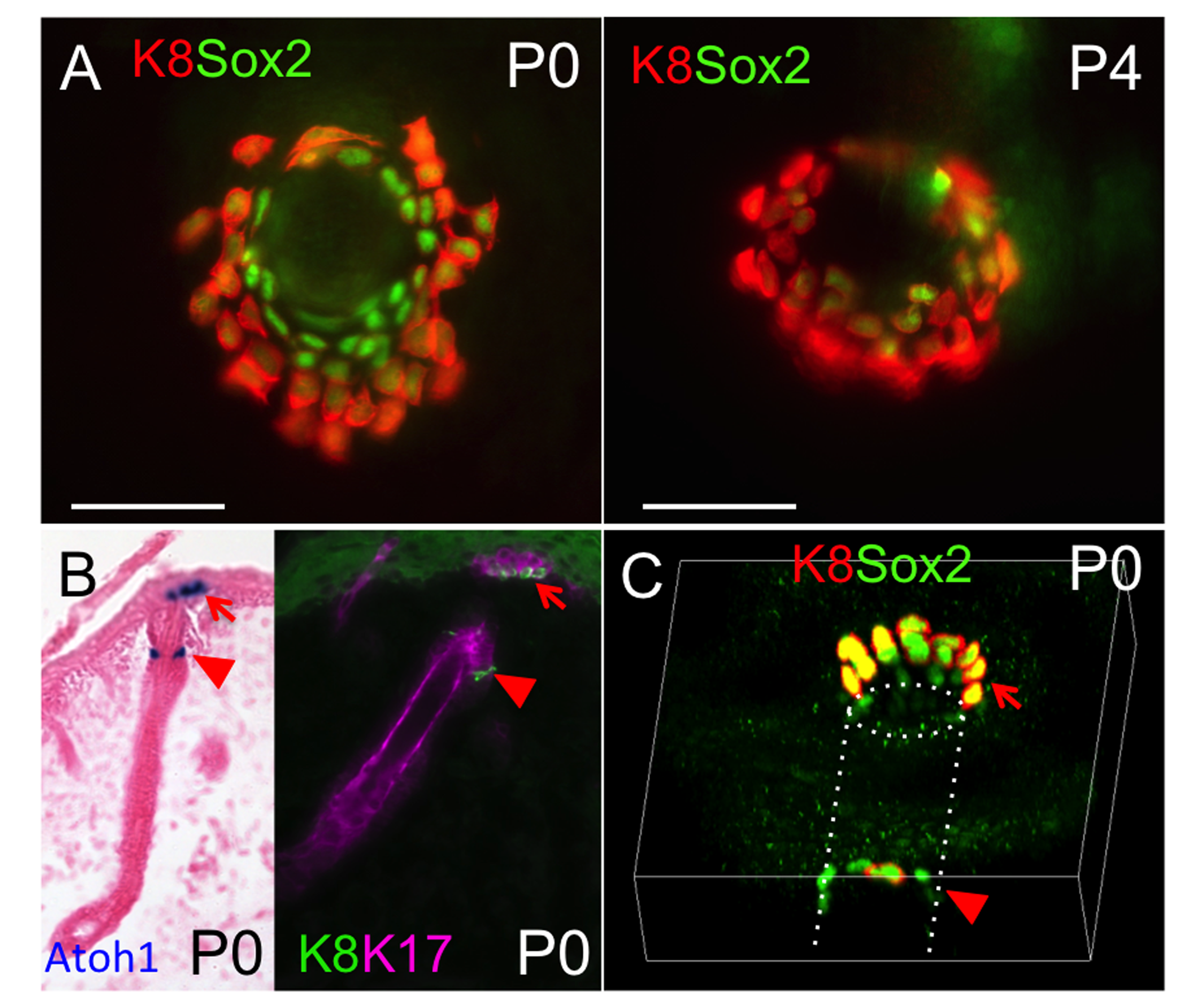

Supplement: S5 Fig — (A) En face image of Sox2 and K8 whole mount staining in wildtype P0 and P4 touch domes. (B) X-gal section staining in Atoh1LacZ/+ skin at P0 and K8 and K17 section staining in wildtype skin at P0. (C) Confocal maximum projection oblique view of Sox2 and K8 whole mount staining in wildtype skin at P0. Arrows, touch dome in epidermis. Arrowheads, MCs in upper hair follicle. Scale bars, 50 μm. (TIF) [file pgen.1006150.s005.tif]

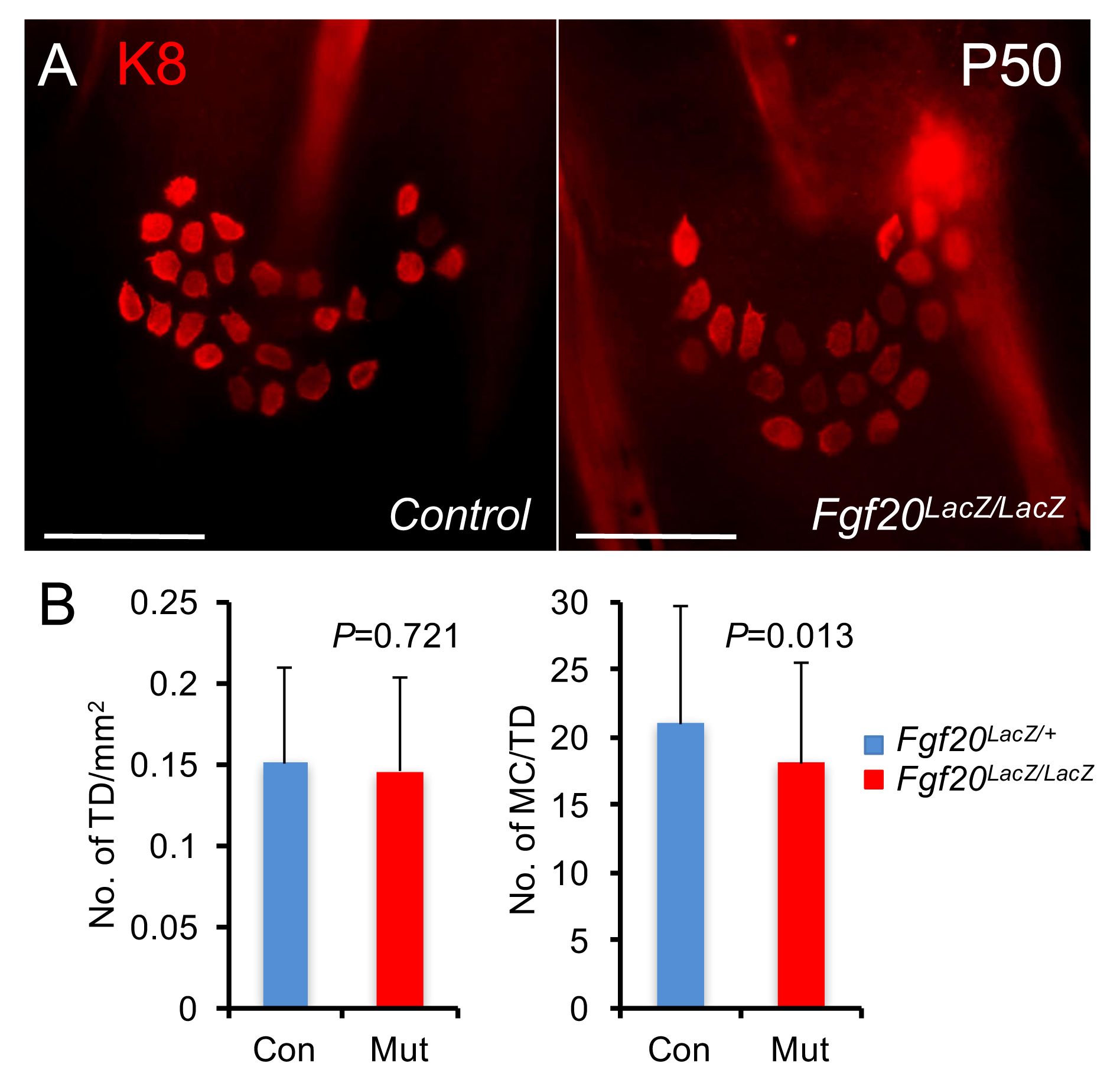

Supplement: S6 Fig — (A) K8 whole mount staining in control (Fgf20LacZ/+) and Fgf20LacZ/LacZ skin at P50. Scale bar, 50 μm. (B) Quantification of TD density per mm2 and MC number per TD in control and Fgf20LacZ/LacZ dorsal trunk skin of adult (P50 –P103) mice. (TIF) [file pgen.1006150.s006.tif]
