# Supplementary material for: A Cascade of Wnt, Eda, and Shh Signaling Is Essential for Touch Dome Merkel Cell Development
Source: PLoS Genet. 2016 Jul 14;12(7):e1006150. doi: 10.1371/journal.pgen.1006150 (PMC4944988; doi:10.1371/journal.pgen.1006150)
Supplement: S1 Table — (PDF) [file pgen.1006150.s007.pdf]

Table S1. Primers used for quantitative RT-PCR.

Gene	Gene ID	Forward Primer	Reverse Primer
Actin	11461	TGGCGCTTTTGACTCAGGAT	GGGATGTTTGCTCCAACCAA
Sox2	20674	GCGGAGTGGAAACTTTTGTCC	CGGGAAGCGTGTACTTATCCTT
Atoh1	11921	ATCCCGTCCTTCAACAACGAC	CTCTCCGACATTGGGAGTCTG
Shh	20423	TGGAAGCAGGTTTCGACTGG	CTCCGGGACGTAAGTCCTTCA
Gli1	14632	CTCGACCTGCAAACCGTAATC	TCCTAAAGAAGGGCTCATGGTA
Edar	13608	ACTCCAACTGTGGTGAGAACG	CGTCGTCTTTAGTGCCGTATC
K8	16691	CAAGGTGGAACTAGAGTCCCG	CTCGTACTGGGCACGAACTTC
Eda	13607	GTGGACGGCACCTACTTCATC	CACCATCTTCACGGCGATTT
